# Late-onset rheumatoid arthritis has a similar time to remission as younger-onset rheumatoid arthritis: results from the Ontario Best Practices Research Initiative

**DOI:** 10.1186/s13075-022-02952-1

**Published:** 2022-11-19

**Authors:** Xiuying Li, Angela Cesta, Mohammad Movahedi, Claire Bombardier

**Affiliations:** 1 Ontario Best Practices Research Initiative, Toronto General Hospital Research Institute, University Health Network, 200 Elizabeth Street, 13EN-224, Toronto, ON M5G 2C4 Canada; 2 Institute of Health Policy, Management, and Evaluation (IHPME), University of Toronto, Toronto, ON Canada; 3 Division of Rheumatology, Mount Sinai Hospital, Toronto, Canada; 4 Department of Medicine, (DOM) and Institute of Health Policy, Management, and Evaluation (IHPME), University of Toronto, Toronto, ON Canada

**Keywords:** Late onset, Rheumatoid arthritis, Time to remission, Prognosis, Treatment regimen

## Abstract

**Background:**

The prevalence of rheumatoid arthritis (RA) in persons 60 years or older is estimated to be 2%. Late-onset rheumatoid arthritis (LORA) is traditionally defined as the onset of RA after the age of 60 years. Compared to younger-onset rheumatoid arthritis (YORA) which occurs before the age of 60 years, LORA has unique characteristics and disease manifestations. To date, few reports have addressed LORA and the prognosis of LORA patients remains unclear. We compared the clinical characteristics, time to remission and treatment regimen at remission between LORA and YORA patients.

**Methods:**

This prospective cohort study used a registry database in Ontario, Canada from 2008 to 2020. Patients were included if they had active rheumatoid arthritis (RA) disease (≥1 swollen joint) and were enrolled within 1 year of diagnosis. LORA was defined as a diagnosis of RA in persons 60 years and older and YORA as a diagnosis of RA in persons under the age of 60. Remission was defined by Disease Activity Score 28 (DAS28) ≤2.6. A multivariable Cox proportional hazards model was used to estimate time to remission.

**Results:**

The study included 354 LORA patients and 518 YORA patients. The mean (standard deviation) baseline DAS28 score was 5.0 (1.3) and 4.8 (1.2) in LORA and YORA patients, respectively (*p*=0.0946). Compared to YORA patients, the hazard ratio for remission in LORA patients was 1.10 (95% confidence interval 0.90 to 1.34 *p*=0.36) after adjusting for other prognostic factors. For patients who reached remission, LORA patients were less likely to be on a biologic or Janus kinase (JAK) inhibitor (16% vs. 27%) and more likely to be on a single conventional synthetic disease-modifying anti-rheumatic drugs (csDMARD) (34% vs. 27%) than YORA patients (*p*=0.0039).

**Conclusion:**

LORA and YORA patients had similar prognosis in terms of time to remission. At remission, LORA patients were more likely to be on a single csDMARD without a biologic or JAK inhibitor.

**Supplementary Information:**

The online version contains supplementary material available at 10.1186/s13075-022-02952-1.

## Introduction

The prevalence of rheumatoid arthritis (RA) in persons 60 years or older is estimated to be 2% [1]. Late-onset rheumatoid arthritis (LORA) is traditionally defined as the onset of RA after the age of 60 years [2]. It is estimated that the incidence and prevalence of RA increase with age up to the age of 80 to 85 years [3, 4]. Therefore, in an ageing population with an increasingly longer life expectancy, the number of LORA patients will continue to increase.

Compared to younger-onset rheumatoid arthritis (YORA), which presents before the age of 60 years, LORA has unique characteristics and disease manifestations such as a higher proportion of males, more comorbidities, less frequent positivity for rheumatoid factor or anti-cyclic citrullinated peptide (anti-CCP) antibody, higher C-reactive protein (CRP), and higher erythrocyte sedimentation rate (ESR) [5].

The prognosis of LORA patients has been unclear in previous studies. Compared to YORA, the prognosis of LORA was better in some studies [6, 7] and worse in others [8–11]. However, knowledge of prognosis is important when counselling a patient with LORA. Clinicians can use this knowledge when weighing the benefits and risks of different RA treatment regimens. This treatment decision is more complex in LORA patients, because age, comorbidities, and frailty can increase the risk of toxicity of disease-modifying anti-rheumatic drugs (DMARDs) [12].

Using data from a large registry of RA patients in Ontario, Canada, we have previously described the clinical characteristics of LORA and YORA patients [13]. The objective of this study was to compare the remission rate and treatment regimen at remission between LORA and YORA patients.

## Methods

### Study design

This was a prospective multicenter cohort study. The Ontario Best Practices Research Initiative (OBRI) is a multicenter provincial registry in Ontario, Canada, that prospectively follows RA patients who are under the routine care of rheumatologists [14]. Patients provided written informed consent prior to enrolment in the registry. Research ethics approval was obtained at the institution (University Health Network Research Ethics Board 07-0729 AE) as well as at each participating site.

### Study population

To be included in the OBRI registry, a patient must be 18 years or older at enrolment, with disease onset after 16 years of age and a rheumatologist-confirmed RA diagnosis.

This study used data from patients who were enrolled in the OBRI registry from January 17, 2008, to January 1, 2020. Patients were included in this study if they were enrolled in the OBRI registry the same or next calendar year relative to their RA diagnosis, not in remission as per the Disease Activity Score 28 joint count (DAS28) score [15] at enrolment and followed for a minimum of 6 months in the OBRI registry.

### Data collection

At each routine clinic visit, the rheumatologist completed a case report form. Data was also collected from the patient via a telephone interview every 6 months. Data collection included age, sex, family history, smoking history, comorbidities, disease activity (DAS28), Health Assessment Questionnaire Disability Index (HAQ DI) [16] and medications.

### Definition of variables

Patients who were diagnosed with RA at the age of 60 years or older were classified as late-onset RA (LORA), whereas patients diagnosed with RA at an age younger than 60 years were classified as younger-onset RA (YORA).

RA medications were classified into conventional synthetic (csDMARD), or biologic/Janus kinase (JAK) inhibitors. The DMARD regimen was classified based on the number of csDMARDs used and the need for biologic or JAK inhibitors.

The primary outcome was time to remission as defined by a DAS28 score of ≤2.6 [15]. Persistent remission was defined as remission for at least in two visits. We also compared the DMARD regimen of LORA and YORA patients when they first reached remission.

### Statistical analysis

Continuous variables were described using mean and standard deviation, or median and interquartile range (IQR) when appropriate. Categorical variables were described using numbers and percentages. Comparison between LORA and YORA patients were done with Student’s *T*-test for continuous variables and chi-squared test for categorical variables.

A Cox proportional hazards model was used to estimate hazard ratio (HR) for time to the first remission defined as DAS28 ≤2.6. In the univariate analysis, potential predictors included age, gender, smoking history, family history, comorbidities, RA disease characteristics, baseline DAS28 score and treatment. Treatment with a biologic or JAK inhibitor was entered as a time-dependent variable. Most of these predictors were selected based on a prior systemic review of important predictors for remission in RA [17]. Selection of predictors for the multivariable Cox proportional hazards model was based on *p*-value <0.2 in the univariate analysis. As well, predictors that did not satisfy the *p*-value <0.2 but were deemed clinically important prognostic factors were also entered into the multivariable model along with LORA.

We performed two sensitivity analyses. First, we performed a subgroup analysis, where only seropositive patients with positive rheumatoid factor or anti-CCP were included in the Cox proportional hazards model for time to remission. Second, we used remission as defined by simplified disease activity index (SDAI) ≤3.3 [18] instead of the DAS28 score as the dependent variable for the Cox proportional hazards model.

All reported confidence intervals (CIs) were two-sided 95% intervals and all tests were two-sided with a *p*<0.05 significance level. All analyses were done using SAS 9.4.

## Results

**Fig. 1:**
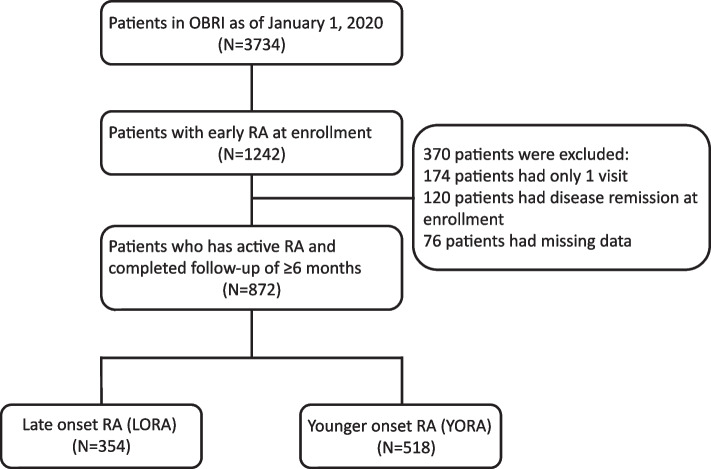
Flow diagram

In total, 872 participants in the OBRI registry had early and active RA. On enrolment, 734 (84%) patients had definite RA based on the American College of Rheumatology / European League Against Rheumatism 2010 criteria [19]. Of the 872 patients, 354 (41%) patients had LORA and 518 (59%) patients had YORA (Fig. 1).

### Baseline characteristics

Baseline characteristics of patients at enrolment are described in Table 1. LORA and YORA patients had a mean age of 69.8 and 47.2 years, respectively (*p*<0.0001). Compared to YORA patients, LORA patients were more likely to be male (34% versus 20%, *p*<0.0001), and less likely to have a positive rheumatoid factor or anti-CCP (63% vs. 75%, *p*=0.0003). LORA patients had a higher number of comorbidities (mean of 1.5 vs. 0.8, *p*<0.0001). Although the DAS28 score was similar between the two groups, LORA patients had significantly higher inflammatory markers (ESR and CRP).

**Table 1:** Baseline characteristics at enrolment^a^

	LORA ***N***=354	YORA N=518	***p***-value
**Sociodemographic characteristics**			
Age (years) mean (SD)	69.8 (6.7)	47.2 (9.7)	<.0001
Female	232 (66%)	414 (80%)	<.0001
RA family history	85 (25%)	116 (23%)	0.5274
Ever smoked	197 (56%)	256 (49%)	0.0706
Post-secondary education	156 (44%)	329 (64%)	<.0001
Treated by an academic rheumatologist	84 (24%)	123 (24%)	0.9956
**Disease characteristics**			
DAS28, mean (SD)	5.0 (1.3)	4.8 (1.2)	0.0946
28 swollen joint count, mean (SD)	6.6 (4.9)	6.2 (4.8)	0.1468
Swollen large joints, mean (SD)	0.7 (1.0)	0.7 (1.1)	0.8276
Swollen small joints, mean (SD)	5.7 (4.8)	5.2 (4.5)	0.1512
28 tender joint count, mean (SD)	7.5 (6.3)	7.8 (6.4)	0.4864
Tender large joints, mean (SD)	1.1 (1.5)	1.3 (1.6)	0.2006
Tender small joints, mean (SD)	5.9 (5.7)	6.2 (5.7)	0.5335
ESR, mm/h, mean (SD)	31.1 (24.0)	23.8 (18.0)	<.0001
CRP, mg/L, mean (SD)	19.7 (26.6)	13.3 (21.3)	0.0003
Physician global assessment (0–10), mean (SD)	4.9 (2.3)	5.2 (2.3)	0.0513
Patient global assessment (0–10), mean (SD)	5.0 (2.7)	5.6 (2.7)	0.0030
Simple Disease Activity Index (SDAI)	27.0 (14.0)	26.6 (13.7)	0.6701
Either anti-CCP or rheumatoid factor positive	219 (63%)	379 (75%)	0.0003
Rheumatoid factor positive	209 (60.8%)	357 (71.5%)	0.0010
Anti-CCP positive	102 (51.8%) *n*=197	200 (63.5%) *n*=315	0.0078
Joint erosion	93 (32%)	75 (18%)	<.0001
Extra-articular features	87 (25%)	95 (18%)	0.0261
Nodule	22 (6%)	27 (5%)	0.5279
Interstitial lung disease	5 (1%)	1 (0.2%)	0.0432
Ocular	0 (0%)	2 (0.4%)	0.5171
Neurologic disease	4 (1%)	1 (0.2%)	0.1645
Sjogren’s	3 (1%)	5 (1%)	1.0000
Other	55 (16%)	59 (11%)	0.0745
Ever tried biologic or JAK inhibitor	12 (3%)	28 (5%)	0.1614
Ever tried csDMARD	170 (48%)	269 (52%)	0.2849
Number of comorbidities, mean (SD)	1.5 (1.3)	0.8 (1.1)	<.0001
Osteoarthritis	130 (37%)	75 (14%)	<.0001
Depression	30 (6%)	65 (13%)	0.0580
Hypertension	155 (44%)	64 (12%)	<.0001
Cardiovascular	88 (25%)	49 (9%)	<.0001
Lung disease (excludes Asthma)	37 (10%)	20 (4%)	0.0001
Liver disease	13 (4%)	9 (2%)	0.0736
Cancer	45 (13%)	15 (3%)	<.0001
**Functional status**			
HAQ-DI (0–3), mean (SD)	1.19 (0.71)	1.16 (0.73)	0.4457
HAQ pain (0–3), mean (SD)	1.37 (0.82)	1.61 (0.80)	0.0001
Morning stiffness last >30 min	167 (47%)	307 (60%)	0.0005
Fatigue (0–10), mean (SD)	4.6 (3.1)	5.5 (3.0)	0.0001
Sleep (0–10), mean (SD)	4.1 (3.3)	4.7 (3.4)	0.0092
Diagnosis from the first symptom years mean (SD)	3.2 (7.1)	2.7 (5.7)	0.3106
How the arthritis affected patients’ ability to participate in normal activities (housework, gardening, childcare, school activities etc.) during the past 3 months (0-10, not effective to completely effective) mean (SD)	5.3 (3.0)	5.7 (2.7)	0.0583
How often the arthritis interfered with how well you get along with others who are close to the patient (1–6 none of time to all of the time) mean (SD)	1.9 (1.1)	2.2 (1.2)	<.0001
**DMARD regimen**			0.0100
Biologic or JAK inhibitor only	4 (1%)	6 (1%)	
Biologic or JAK inhibitor with csDMARD	7 (2%)	34 (7%)	
csDMARD monotherapy	180 (51%)	220 (42%)	
Combination csDMARD	141 (40%)	225 (43%)	
No DMARD	22 (6%)	33 (6%)	
**Other RA treatment**			
Oral glucocorticoid	82 (23%)	75 (14%)	0.0010
Mean dose of prednisone mg/day	10.8 (6.0)	11.1 (7.0)	0.2271
NSAIDs	115 (32%)	215 (42%)	0.0070

*CRP* C-reactive protein, *csDMARD* conventional synthetic disease-modifying anti-rheumatic drug, *DMARD* disease-modifying anti-rheumatic drug, *ESR* erythrocyte sedimentation rate, *HAQ* health assessment questionnaire, *HAQ-DI* health assessment questionnaire disability index, *JAK* Janus kinase, *NSAID* non-steroidal anti-inflammatory drug, *RA* rheumatoid arthritis, *SD* standard deviation

^a^The numbers describe *N* (%) unless specified otherwise

In terms of treatment at enrolment, LORA patients were less likely to be on a biologic or JAK inhibitor (3% vs. 8%, *p*=0.0100) or non-steroidal anti-inflammatory drugs (NSAID) (32% vs. 42%, *p*=0.0070). Also, a significantly higher proportion of LORA patients were taking an oral glucocorticoid (23% vs. 14%, *p*=0.0010).

### Time to remission

**Fig. 2:**
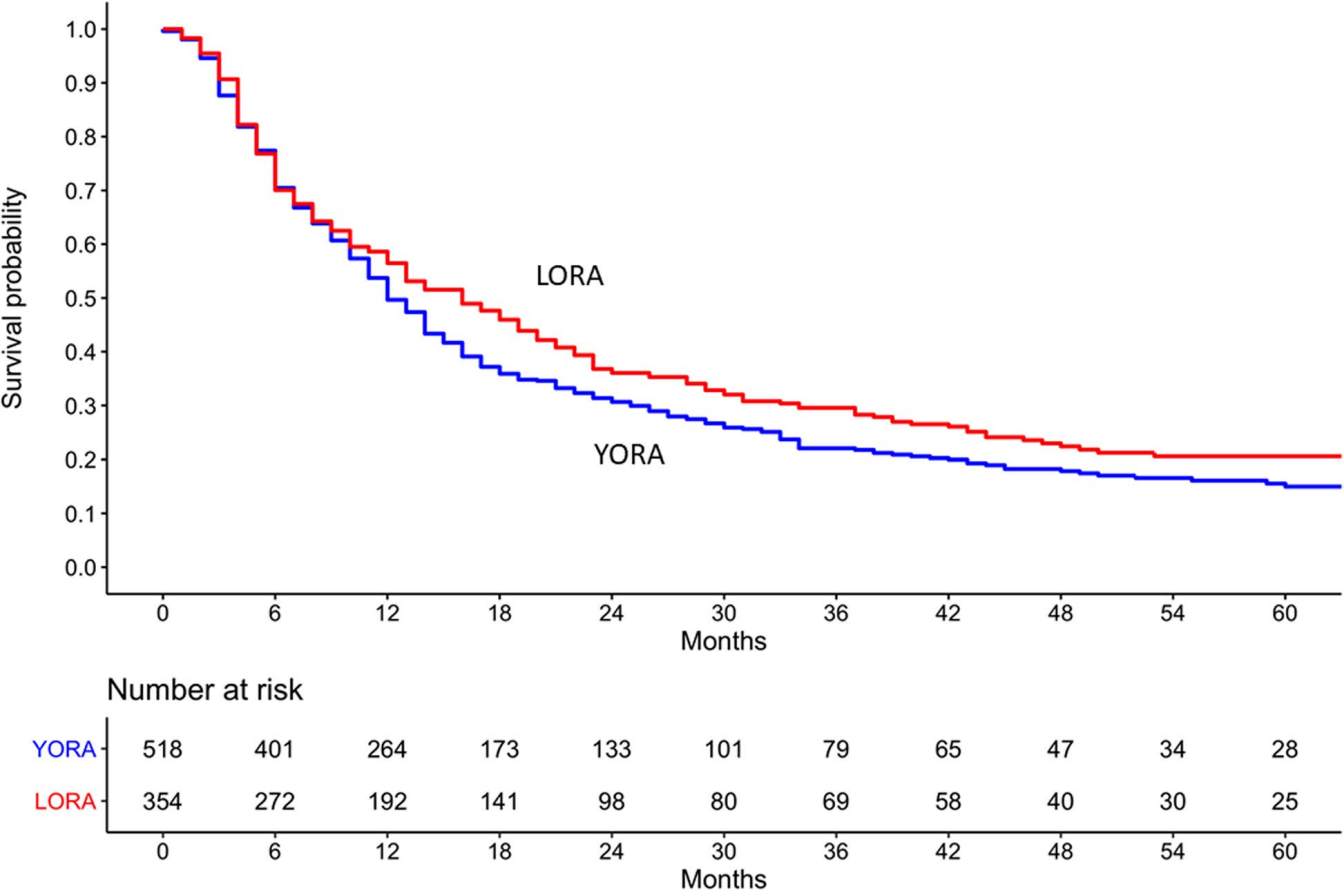
Kaplan Meier survival curve of time to remission

The median (IQR) follow-up time to remission was 12.2 (IQR 6.0 to 25.5) months for all patients, 13.2 (IQR 6.1 to 26.9) months for LORA patients, and 11.9 (IQR 6.0 to 24.3) months for YORA patients. The mean (SD) follow-up time to remission was 19.5 (19.9) months for all patients, 20.6 (20.5) months for LORA patients and 18.7 (19.4) months for YORA patients. The decrease in DAS28 score, over time as compared to the baseline for each patient, is described in Table 2. The survival curves of LORA and YORA patients for active disease not at remission are shown in Fig. 2. At the end of follow-up, 72% of LORA patients and 78% of YORA patients reached remission. Furthermore, 54% of LORA patients and 48% of YORA patients reached persistent remission.

**Table 2:** Decrease in DAS28 and components at follow-up compared to baseline for each individual patient

Decrease compared to baseline	LORA patients	YORA patients
6 months, mean (SD)	*n*=315	*n*=443
DAS28	1.6 (1.7)	1.5 (1.5)
Swollen joint count (0–28)	3.9 (5.1)	3.9 (4.8)
Tender joint count (0–28)	4.1 (6.7)	4.0 (6.7)
ESR mm/h	9.3 (23.0)	8.0 (16.1)
Patient global assessment (0–10)	2.1 (3.4)	1.8 (3.1)
12 months, mean (SD)	*n*=280	*n*=398
DAS28	1.9 (1.8)	1.9 (1.7)
Swollen joint count (0–28)	4.3 (5.2)	4.3 (5.1)
Tender joint count (0–28)	4.8 (6.9)	4.8 (6.9)
ESR mm/h	11.8 (23.5)	10.3 (17.8)
Patient global assessment (0–10)	2.2 (3.3)	2.2 (3.3)
18 months, mean (SD)	*n*=239	*n*=352
DAS28	1.9 (1.8)	1.8 (1.7)
Swollen joint count (0–28)	4.3 (5.3)	4.0 (5.4)
Tender joint count (0–28)	4.8 (7.0)	4.5 (6.8)
ESR mm/h	12.5 (23.1)	10.2 (17.8)
Patient global assessment (0–10)	2.3 (3.3)	2.2 (3.3)
24 months, mean (SD)	*n*=208	*n*=301
DAS28	2.3 (1.8)	1.9 (1.7)
Swollen joint count (0–28)	5.1 (5.5)	4.5 (5.3)
Tender joint count (0–28)	6.0 (7.3)	4.9 (7.5)
ESR mm/h	15.7 (25.4)	10.9 (18.7)
Patient global assessment (0–10)	2.5 (3.3)	2.0 (3.2)

*SD* standard deviation

Univariate and multivariable Cox proportional hazard model predicting time to remission is shown in Table 3. After adjusting for significant predictors, the adjusted hazard ratio for LORA was 1.10 (95% CI 0.90 to 1.34, *p*=0.36).

**Table 3:** Cox proportional hazards model predicting time to remission

Baseline characteristics	Univariate		Multivariable	
	HR (95% CI)	*p*-value	HR (95% CI)	*p*-value
**Sociodemographic**				
Female gender	0.71 (0.60–0.84)	<.0001	0.87 (0.70–1.09)	0.2256
Post-secondary education	1.26 (1.08–1.47)	0.0039	1.04 (0.87–0.70)	0.6744
Ever smoked	0.87 (0.75–1.02)	0.076	0.93 (0.77–1.12)	0.4269
RA family history	0.89 (0.74–1.07)	0.2176	0.87 (0.70–1.70)	0.1817
**Disease characteristics**				
Positive rheumatoid factor	1.01 (0.85–1.19)	0.9182	0.94 (0.78–1.14)	0.5381
HAQ-DI	0.62 (0.55–0.69)	<.0001	0.71 (0.61–0.84)	<.0001
Morning stiffness (>30 min)	0.71 (0.61–0.83)	<.0001	0.89 (0.73–1.08)	0.2366
Joint erosion	0.94 (0.77–1.14)	0.5224	0.87 (0.70–1.08)	0.1954
DAS28	0.77 (0.72–0.82)	<.0001	0.88 (0.80–0.96)	0.0048
Extra-articular features	0.95 (0.78–1.14)	0.5534		
Number of comorbidities	0.83 (0.77–0.88)	<.0001	0.88 (0.81–0.95)	0.0022
Osteoarthritis	0.93 (0.78–1.12)	0.4497		
**Treatment**				
Oral glucocorticoid	1.06 (0.86–1.30)	0.5800		
Biologic or JAK inhibitor (time variant)	0.86 (0.71–1.03)	0.09	1.53 (0.63–3.69)	0.3485
**LORA**	0.83 (0.71–0.97)	0.0194	1.10 (0.90–1.34)	0.3593

*CI* confidence interval, *DAS28* Disease Activity Score 28 joint count, *JAK* Janus kinase, *HR* hazard ratio, *HAQ-DI* health assessment questionnaire disability index, *LORA* late-onset rheumatoid arthritis, *RA* rheumatoid arthritis

As a sensitivity analysis, the multivariable Cox proportional hazards model for the subgroup of seropositive patients with a positive rheumatoid factor or anti-CCP is shown in Supplementary Materials Table S1. In another sensitivity analysis, the multivariable Cox proportional hazards model using remission as defined by SDAI≤3.3 is shown in Supplementary Materials Table S2. Both sensitivity analyses showed consistent results of similar remission rates between LORA and YORA patients.

### Patients at remission

The DAS28 components and medication regimen of LORA and YORA patients when they first reached remission are shown in Table 4. Compared to YORA patients, LORA patients were less likely to be on a biologic or JAK inhibitor (16% vs. 27%) and more likely to be on a single csDMARD (34% vs. 27%, *p*=0.0039). Furthermore, a significant proportion of LORA patients were on oral glucocorticoids at the time of remission (27% vs. 13%, *p*<0.0001).

**Table 4:** Clinical characteristics and treatment regimen of patients when they first reached remission^a^

	LORA (***N***=254)	YORA (***N***=405)	***P***-value
**DAS28 components**			
Tender joint count, mean (SD)	0.6 (1.3)	0.5 (1.0)	0.1422
Swollen joint count, mean (SD)	0.9 (1.5)	0.8 (1.5)	0.6349
ESR, mean (SD)	10.2 (8.8)	7.8 (0.4)	0.3834
CRP, mean (SD)	4.4 (9.2)	3.9 (5.9)	0.4824
Patient global assessment, mean (SD)	2.0 (1.8)	2.2 (2.0)	0.3465
Physician global assessment, mean (SD)	1.3 (1.3)	1.4 (1.3)	0.3611
**HAQ**			
HAQ-DI mean (SD)	0.8 (0.7)	0.6 (0.7)	0.0281
HAQ pain mean (SD)	0.8 (0.8)	0.9 (0.8)	0.0450
**DMARD regimen**			0.0039
Biologic or JAK inhibitor with or without csDMARD	41 (16%)	110 (27%)	
Combination csDMARD	115 (45%)	169 (42%)	
csDMARD monotherapy	86 (34%)	110 (27%)	
**Other RA treatment**			
NSAIDS	44 (17%)	90 (22%)	0.1283
Oral glucocorticoid	68 (27%)	51 (13%)	<.0001
Mean (SD) dose of prednisone mg/day	10.2 (9.3)	8.3 (7.6)	0.2347

*CRP* C-reactive protein, *csDMARD* conventional synthetic disease-modifying anti-rheumatic drug, *ESR* erythrocyte sedimentation rate, *HAQ* health assessment questionnaire, *HAQ-DI* health assessment questionnaire disability index, *NSAID* non-steroidal anti-inflammatory drug, *RA* rheumatoid arthritis, *SD* standard deviation

^a^The numbers describe *N* (%) unless specified otherwise

### Adverse events due to treatment

The rate of serious infections and new cancers stratified by use of biologic or JAK inhibitor are described for LORA and YORA patients in Supplementary Materials Table S3. LORA patients had consistently higher rates of serious infections and new cancers than YORA patients irrespective of biologics or JAK inhibitor use (Supplementary Materials Table S3).

## Discussion

In this cohort study, LORA patients had a similar time to remission to YORA patients after adjusting for other prognostic factors (HR of 1.10, 95% CI 0.90 to 1.34). For those patients who reached remission, LORA patients were more likely to be on a less intensive DMARD regimen such as using a single csDMARD without a biologic or JAK inhibitor.

In our study, LORA patients had a higher proportion of males, a lower proportion of RF or anti-CCP positivity, and higher inflammatory markers compared to YORA patients. These findings were similar to other published studies [5, 8, 13].

Previous studies have shown conflicting results on the prognosis of LORA patients compared to YORA patients. LORA patients had a better prognosis in some older studies [6, 7] and a worse prognosis in more recent studies [8–11]. For example, in a large multicenter French cohort study of 698 patients with early RA, 118 LORA patients (age >60 years) had an adjusted odds ratio of 0.33 (95% CI 0.16 to 0.71) for remission at 1 year using SDAI, compared to YORA patients (age <45 years), suggesting a worse prognosis for LORA [10]. Whereas, in a US cohort study of 422 patients with RA onset within 2 years, the remission rates using the American Rheumatism Association (ARA) criteria in 214 LORA patients (age ≥65 years), and 186 YORA patients (age <65 years) were 46% and 20%, respectively, suggesting a better prognosis for LORA [6].

For studies that reported disease activity based on DAS28, the mean area under the curve was 47.7 in LORA patients versus 44.8 in YORA patients (*P*<0.01) at 1 year in a cohort of 750 RA patients [8]. In another study, LORA had a lower response to therapy based on DAS28 <3.2 at 6 months with an odds ratio of 0.28 (95% CI 0.08 to 0.98) in 140 patients with early RA [11]. Finally, in a cohort of 229 patients with early RA, although DAS28 was higher at baseline in LORA (mean of 5.0 vs. 4.0 p<0.001), the DAS28 was not significantly different between the two groups at the end of the 2-year follow-up (mean of 2.5 vs. 2.3 *p*=0.07) [9]. Our study results are consistent with this last study [9], which had a large sample of patients with early RA with a long follow-up.

In contrast to these studies, our study results provide an estimate showing a similar prognosis between LORA and YORA patients. The different results may be related to differences in the definition of LORA, the time point with respect to the disease course at enrolment, criteria for remission, the geographic location and sample size. Compared to other studies, our study defined LORA as being a diagnosis in the same or next calendar year and a widely accepted remission criteria based on DAS28. As well, our study has a larger sample size than the aforementioned studies allowing for a more precise estimate. The use of different measures for disease activity and remission makes comparison difficult across studies. The most commonly used measures of disease activity include DAS28, Clinical Disease Activity Index (CDAI) and SDAI [20]. The DAS28, CDAI and SDAI cut-offs do not translate into the same clinical information [20]. Even CDAI and SDAI had significant disagreements when applied to the same patients [20]. Of these three composite measures, the DAS28 is the oldest instrument that has been extensively validated and most widely used in clinical practice as well as research [20–22].

The results of this study have important implications to the management of LORA. When deciding on the initial treatment regimen for LORA, it is likely not necessary to start combination DMARDs or a biologic/JAK inhibitor, because many LORA patients were able to reach remission while on csDMARD. This validates the current practice pattern where LORA patients are typically not treated with combination DMARD or a biologic [23, 24]. However, the goal of treatment for LORA should be the same for YORA, given that LORA patients were just as likely as YORA patients to reach remission on follow-up in our study. Therefore, if LORA patients are still not at remission at follow-up, treatment should be escalated in the same aggressive manner as YORA patients so that they may reach remission. For example, in a study of 197 patients with LORA, patients that adhered to a treat-to-target strategy targeting low disease activity with escalation of therapy were more likely to have sustained remission by SDAI (42.2% vs. 14.5% *p*<0.001) as well as less progression of joint destruction and better physical function during 3 years of follow-up [25]. Based on observational cohort studies and randomized controlled trials, the effectiveness and safety of biologic DMARDs are likely similar in elderly patients [25]. Therefore, clinicians should not hesitate to escalate and add a biologic or JAK inhibitor for LORA patients who have been treated with csDMARDs and have not reached remission.

A higher proportion of LORA patients were initiated on glucocorticoids in our study. LORA patients may have more active disease at baseline based on higher inflammatory markers and joint erosions, prompting the rheumatologist to treat with glucocorticoid for early disease control. Interestingly, a higher proportion of LORA patients were maintained on glucocorticoids at remission. Similarly, in a large observational cohort of 4202 patients, prednisone use was much higher in LORA patients than in YORA patients (41.0% vs. 37.6% *P*=0.025) [23]. Prolonged glucocorticoid therapy can have potential adverse effects [26]. This presents an opportunity to improve the care of LORA. Once LORA patients reach remission, the rheumatologist should re-evaluate the treatment regimen and readjust the regimen if necessary to make it safer in the long term.

Our study has several strengths. First, this is one of the largest studies to date describing the prognosis of LORA patients over time. The large sample size allowed for more precise estimates. Second, this study has detailed data on clinical characteristics, treatment and outcome over a long period of follow-up. The data collection is prospective, rigorous and complete.

Our study also has several weaknesses. First, there may be residual confounding due to the observational nature of the study. However, we used a Cox proportional hazards model to adjust for other important prognostic factors. The significant predictors and correlation with prognosis in the final multivariable models were consistent with the important predictors of remission in a previous systematic review [17]. One significant predictor was the number of comorbidities, which may bias the assessment of disease activity. It is not possible to ascertain how much of disease activity is attributable to comorbidities versus RA. Thus, patients may have persistent symptoms due to comorbidities when their RA is actually in remission. This would underestimate the remission rate in LORA patients who had more comorbidities. Second, in an ideal inception cohort, all patients should be recruited at the onset of symptoms. This is not feasible in a cohort study. Nevertheless, our criteria of enrolment date at the same or next calendar year relative to the diagnosis of RA was more stringent than previous studies [6, 27] and should be representative of patients with early and active RA. Third, there may be variations in the treatment of LORA patients across centres and over time. In the 2011/2012 Canadian Rheumatology Association guidelines on pharmacotherapy of RA [15], there is no special consideration for LORA and this serves as general guidance for Canadian rheumatologists. Our study findings are representative of real-world data on the management of LORA patients.

## Conclusions

Our study findings suggest that LORA patients have a similar prognosis as YORA patients, however, LORA patients who reached remission were less likely to be on combination DMARDs or a biologic/JAK inhibitor. This suggests that LORA patients likely do not require combination DMARD or biologic on initiation. Future studies should evaluate if a standardized treatment protocol tailored to LORA patients improves the safety of RA treatment and remission rate.

## Supplementary Information

**Additional file 1: Table S1.** Cox proportional hazards model predicting time to remission within subgroup of seropositive (positive rheumatoid factor or anti-CCP) patients (*N*=598). **Table S2.** Cox proportional hazards model predicting time to remission based on SDAI criteria of ≤3.3 (*N*=748). **Table S3.** Number of patients who experienced a serious infection, a new cancer or an adverse event during follow-up.

## Data Availability

The data underlying this article will be shared on reasonable request to the corresponding author.

## Abbreviations

RA: Rheumatoid arthritis
LORA: Late-onset rheumatoid arthritis
YORA: Younger-onset rheumatoid arthritis
Anti-CCP: Anti-cyclic citrullinated peptide
CRP: C-reactive protein
ESR: Erythrocyte sedimentation rate
DMARD: Disease-modifying anti-rheumatic drug
CsDMARD: Conventional synthetic disease-modifying anti-rheumatic drug
OBRI: Ontario Best Practices Research Initiative
DAS28: Disease Activity Score 28 joint count
SDAI: Simplified disease activity index
CDAI: Clinical Disease Activity Index
HAQ-DI: Health Assessment Questionnaire Disability Index
JAK: Janus kinase
NSAID: Non-steroidal anti-inflammatory drug
