# Supplementary material for: Late-onset rheumatoid arthritis has a similar time to remission as younger-onset rheumatoid arthritis: results from the Ontario Best Practices Research Initiative
Source: Arthritis Res Ther. 2022 Nov 19;24:255. doi: 10.1186/s13075-022-02952-1 (PMC9675233; doi:10.1186/s13075-022-02952-1)
Supplement: Supplementary file 1 — Additional file 1: Table S1. Cox proportional hazards model predicting time to remission within subgroup of seropositive (positive rheumatoid factor or anti-CCP) patients (N=598). Table S2. Cox proportional hazards model predicting time to remission based on SDAI criteria of ≤3.3 (N=748). Table S3. Number of patients who experienced a serious infection, a new cancer or an adverse event during follow-up. [file 13075_2022_2952_MOESM1_ESM.docx]

Supplementary Materials

Table S1. Cox proportional hazards model predicting time to remission within subgroup of seropositive (positive rheumatoid factor or anti-CCP) patients (N=598)

| **Baseline characteristics** | **Multivariable** |  |
| --- | --- | --- |
| **Sociodemographic** | HR (95% CI) | p-value |
| Female gender | 0.97 (0.75-1.26) | 0.8320 |
| Post-secondary education | 0.96 (0.78-1.19) | 0.7178 |
| Ever smoked | 1.05 (0.84-1.31) | 0.6876 |
| RA family history | 0.85 (0.66-1.09) | 0.2072 |
| **Disease characteristics** |  |  |
| Positive rheumatoid factor | 0.94 (0.78-1.14) | 0.5381 |
| HAQ-DI | 0.74 (0.61-0.90) | 0.0019 |
| Morning stiffness (>30 mins) | 0.87 (0.69-1.10) | 0.2552 |
| Joint erosion | 0.86 (0.67-1.10) | 0.2228 |
| DAS28 | 0.87 (0.79-0.96) | 0.0051 |
| Number of comorbidities | 0.85 (0.77-0.94) | 0.0015 |
| **Treatment** |  |  |
| Biologic or JAK inhibitor (time variant) | 1.03 (0.80-1.33) | 0.8023 |
| **LORA** | 1.05 (0.83-1.33) | 0.6573 |

DAS28 = Disease Activity Score 28 joint count; JAK = Janus kinase (JAK) inhibitors; HAQ DI = health assessment questionnaire disability index; LORA = late onset rheumatoid arthritis; RA = rheumatoid arthritis

Table S2. Cox proportional hazards model predicting time to remission based on SDAI criteria of ≤3.3 (N=748)

| **Baseline characteristics** | **Multivariable** |  |
| --- | --- | --- |
| **Sociodemographic** | HR (95% CI) | p-value |
| Female gender | 0.84 (0.66-1.07) | 0.1557 |
| Post-secondary education | 1.38 (1.09-1.75) | 0.0071 |
| Ever smoked | 1.15 (0.91-1.45) | 0.2328 |
| RA family history | 0.95 (0.72-1.25) | 0.6919 |
| **Disease characteristics** |  |  |
| Positive rheumatoid factor | 1.02 (0.80-1.30) | 0.8691 |
| HAQ-DI | 0.60 (0.49-0.73) | <.0001 |
| Morning stiffness (>30 mins) | 0.84 (0.66-1.07) | 0.1557 |
| Joint erosion | 0.88 (0.67-1.15) | 0.3424 |
| SDAI | 1.01 (1.00-1.02) | 0.1031 |
| Number of comorbidities | 0.78 (0.70-0.88) | <.0001 |
| **Treatment** |  |  |
| Biologic or JAK inhibitor (time variant) | 0.88 (0.67-1.15) | 0.3434 |
| **LORA** | 1.23 (0.96-1.57) | 0.1046 |

SDAI = Simple Disease Activity Index; JAK = Janus kinase (JAK) inhibitors; HAQ DI = health assessment questionnaire disability index; LORA = late onset rheumatoid arthritis; RA = rheumatoid arthritis

Table S3. Number of patients who experienced a serious infection, a new cancer or an adverse event during follow-up

|  | LORA  N=354 | YORA  N=518 | P value |
| --- | --- | --- | --- |
| Serious infections* per 100 patient-year  -Tried biologic/JAK inhibitor  -Never tried biologic/JAK inhibitor | 6.5  9.3  4.5 | 3.8  8.1  2.8 | 0.0050 |
| New cancer per 100 patient-year  -Tried biologic/JAK inhibitor  -Never tried biologic/JAK inhibitor | 2.5  2.4  2.5 | 0.5  0.8  0.6 | 0.0006 |
| Adverse event requiring discontinuation of biologic/JAK inhibitor n (%) | 13/74  (17.6%) | 18/140  (12.9%) | 0.3188 |

* Serious infection defined as requiring hospitalization and / or intravenous antimicrobial
